# Sigmoid volvulus revealing hidden schistosomiasis: a case report and literature review

**DOI:** 10.1093/jscr/rjaf676

**Published:** 2025-08-29

**Authors:** Faten Limaiem, Zied Hadrich, Mohamed Hajri, Taher Laabidi, Aziz Atallah, Hafedh Mestiri

**Affiliations:** Faculty of Medicine of Tunis, University of Tunis El Manar, 15, Djebel Lakhdhar Street- 1007 Bab Saadoun, Tunis, Tunisia; Pathology Department, Hospital Mongi Slim La Marsa, Sidi Daoued - 2046, Tunis, Tunisia; Faculty of Medicine of Tunis, University of Tunis El Manar, 15, Djebel Lakhdhar Street- 1007 Bab Saadoun, Tunis, Tunisia; Department of Visceral Surgery, Hospital Mongi Slim La Marsa, Sidi Daoued 2046, Tunis, Tunisia; Faculty of Medicine of Tunis, University of Tunis El Manar, 15, Djebel Lakhdhar Street- 1007 Bab Saadoun, Tunis, Tunisia; Department of Visceral Surgery, Hospital Mongi Slim La Marsa, Sidi Daoued 2046, Tunis, Tunisia; Faculty of Medicine of Tunis, University of Tunis El Manar, 15, Djebel Lakhdhar Street- 1007 Bab Saadoun, Tunis, Tunisia; Department of Visceral Surgery, Hospital Mongi Slim La Marsa, Sidi Daoued 2046, Tunis, Tunisia; Faculty of Medicine of Tunis, University of Tunis El Manar, 15, Djebel Lakhdhar Street- 1007 Bab Saadoun, Tunis, Tunisia; Department of Visceral Surgery, Hospital Mongi Slim La Marsa, Sidi Daoued 2046, Tunis, Tunisia; Faculty of Medicine of Tunis, University of Tunis El Manar, 15, Djebel Lakhdhar Street- 1007 Bab Saadoun, Tunis, Tunisia; Department of Visceral Surgery, Hospital Mongi Slim La Marsa, Sidi Daoued 2046, Tunis, Tunisia

**Keywords:** volvulus, schistosomiasis, sigmoid, surgery, pathology, case report

## Abstract

Sigmoid volvulus is an uncommon cause of intestinal obstruction, typically affecting older adults. Its association with chronic schistosomiasis is exceptionally rare, and the causal relationship remains unclear. We report a case of sigmoid volvulus in a 22-year-old male immigrant from Guinea, which led to the incidental diagnosis of chronic *Schistosoma mansoni* infection. The patient presented with abdominal pain, distension, and constipation. Imaging confirmed sigmoid volvulus, and after failed endoscopic decompression, he underwent sigmoid colectomy. Histopathological examination revealed *Schistosoma* eggs with granulomatous inflammation, confirmed by stool analysis. The patient was treated with praziquantel and recovered uneventfully. This case highlights the diagnostic challenges of schistosomiasis in non-endemic regions, where it may remain undetected until complications arise. Surgeons in such areas should consider schistosomiasis in patients from endemic regions, even when symptoms are nonspecific. Histopathology remains critical for diagnosis, and early treatment can prevent long-term sequelae.

## Introduction

Sigmoid volvulus is a relatively uncommon type of intestinal obstruction characterized by the twisting of the sigmoid colon around its own base [1, 2]. It accounts for 10%–13% of cases in most reported series and is more frequently observed in older adults, with a mean age of 70 years at presentation [1, 2]. Concomitant volvulus of the large bowel and chronic schistosomiasis is particularly rare and the causal link between these two conditions has not been conclusively established [3–5]. Herein, we report a case of sigmoid volvulus unveiling chronic schistosomiasis in a young male immigrant from Guinea. The aim of this case report is to highlight the diagnostic challenges and clinical implications of schistosomiasis in non-endemic regions, where its presence can be easily overlooked. This case report adheres to the SCARE Criteria [6].

## Case presentation

A previously healthy 22-year-old male from Guinea presented to our institution with a 48-h history of progressive abdominal pain and complete cessation of bowel movements. On admission, physical examination revealed a moderately distended abdomen with tenderness on deep palpation and diminished bowel sounds. Vital signs were stable with blood pressure of 120/70 mmHg, pulse rate of 80 bpm, and normal body temperature (37.1°C). Digital rectal examination demonstrated an empty rectal vault with normal sphincter tone, and a reducible right inguinal hernia was noted.

Initial laboratory investigations showed normal hematologic parameters (white blood cells 8200/mm^3^, hemoglobin 14.1 g/dL) and only mildly elevated inflammatory markers (C-reactive protein 1.74 mg/L). Comprehensive metabolic panel revealed normal hepatic function (total bilirubin 0.7 mg/dL, aspartate aminotransferase 39 U/L, alanine aminotransferase 37 U/L) and renal function (creatinine 88 μmol/L, urea 5 mmol/L).

**Figure 1:**
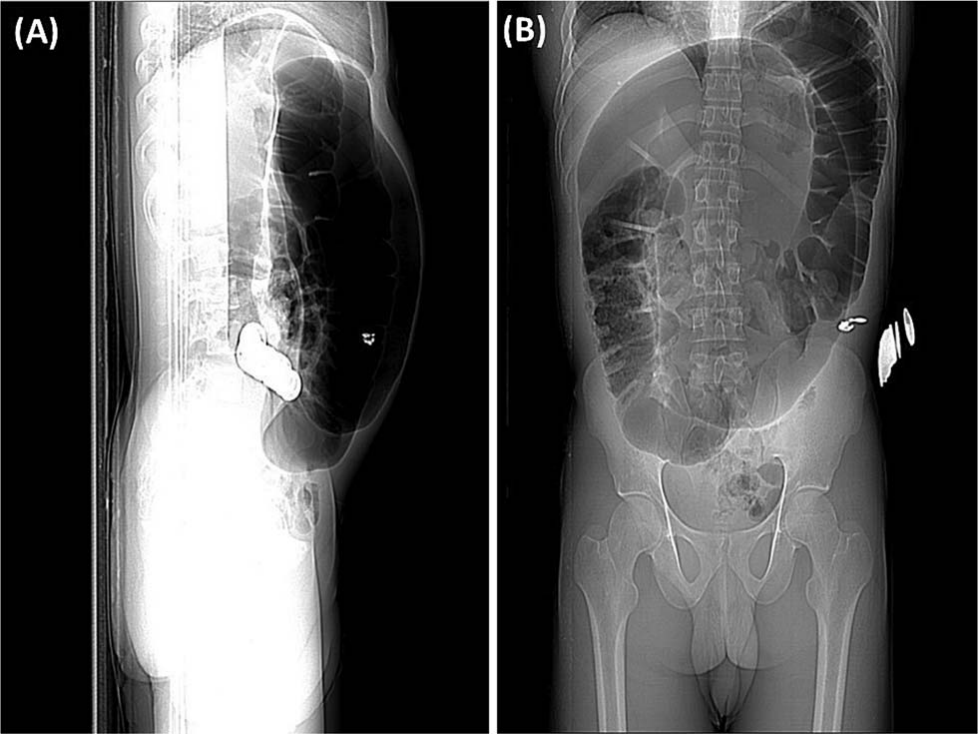
(A) The preliminary view on abdominal CT (scout view) revealed a distended sigmoid loop exhibiting an inverted U-shape, commonly recognized as the coffee bean sign. (B) The preliminary view on abdominal CT (scout view) showed dilated bowel loops.

**Figure 2:**
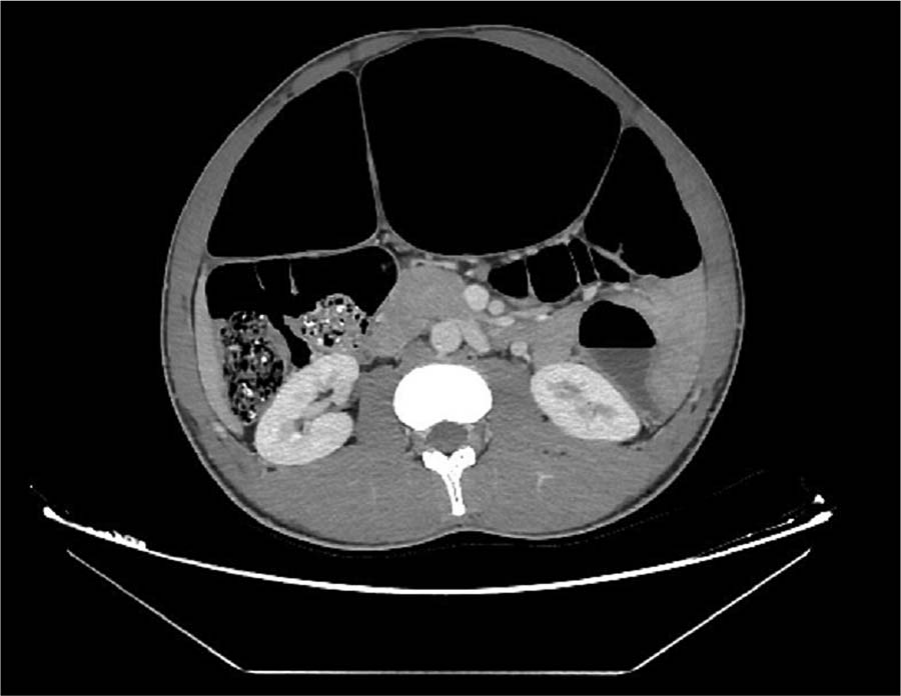
Axial abdominal CT scan revealed dilated gas-filled loops with lack of the haustration.

**Figure 3:**
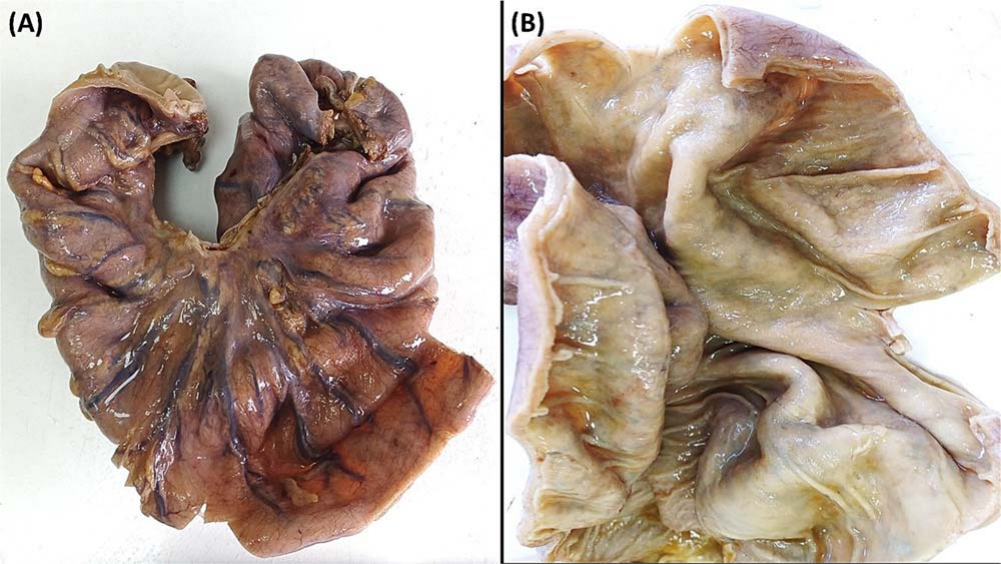
(A) The sigmoid colon, upon gross examination measured 30 cm in length with a maximum diameter of 10 cm. (B) Upon dissection, the colonic mucosa appeared flattened, revealing hemorrhagic spots, signs of vascular congestion, and evident edema.

**Figure 4:**
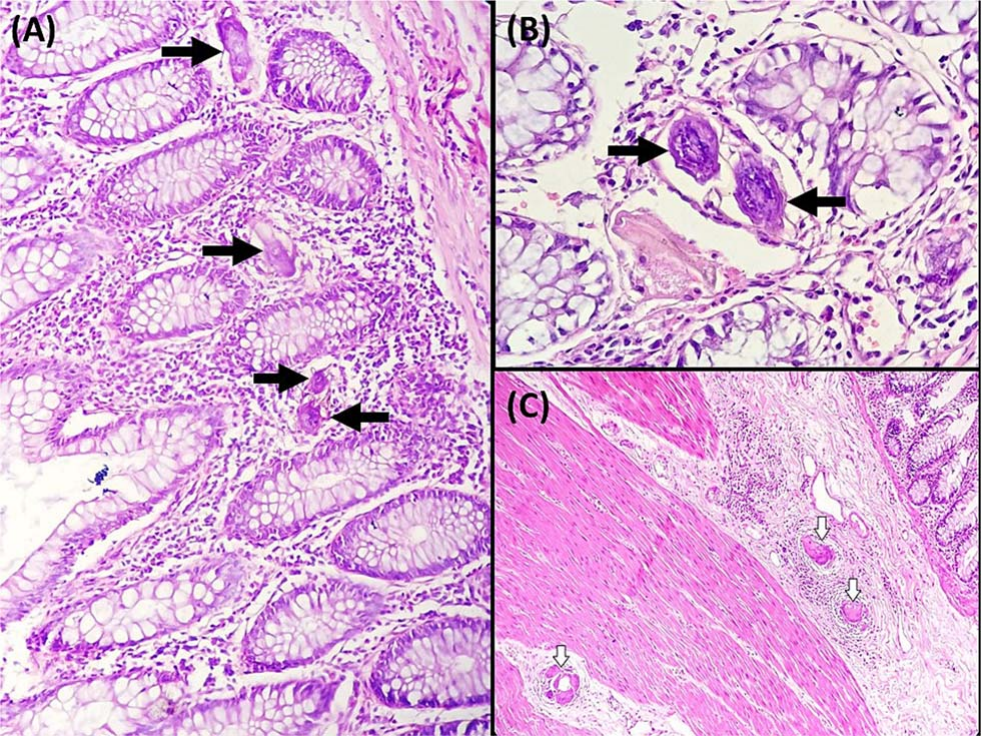
(A) Ovoidal schistosoma eggs surrounded by a polymorphous inflammatory infiltrate (black arrow), (Hematoxylin and eosin, magnification × 200). (B) Microscopic examination reveals Schistosoma eggs which are elongate (Hematoxylin and eosin, magnification × 400). (C) Microscopic analysis of the sigmoid colon revealed the presence of multiple foreign body granulomas within the submucosa and muscular layer (white arrows) (Hematoxylin and eosin, magnification × 100).

**Figure 5:**
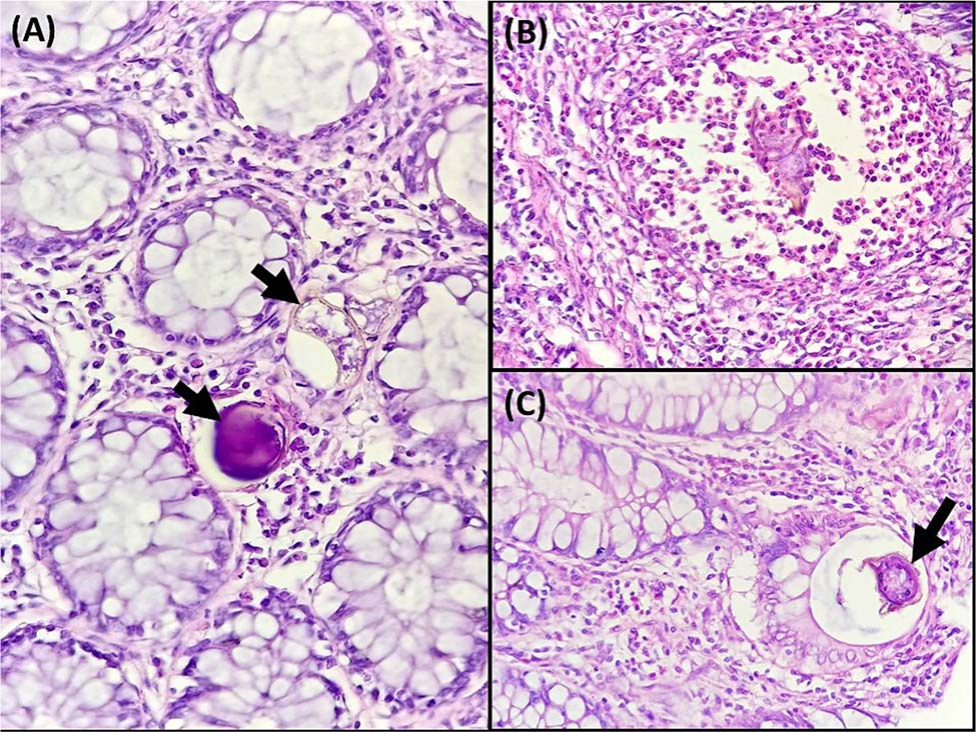
(A) Microscopic analysis of the sigmoid colon revealed calcified schistosomal eggs in the mucosa (black arrow) (Hematoxylin and eosin, magnification ×400). (B) Microscopic examination revealed a polymorphous inflammatory infiltrate with numerous eosinophils surrounding a schistosoma egg (Hematoxylin and eosin, magnification ×400). (C) Microscopic analysis of the sigmoid colon revealed ovoidal schistosoma egg within the crypt lumen (black arrow) (Hematoxylin and eosin, magnification × 400).

Abdominal radiography demonstrated classic features of sigmoid volvulus including dilated colonic loops, multiple air-fluid levels, and the characteristic coffee bean sign with absence of rectal gas. Computed tomography (CT) imaging (Figs 1 and 2) confirmed the diagnosis, revealing large gas-filled loops (maximum diameter 100 mm) without haustration and the typical mesenteric whirl sign, consistent with closed-loop obstruction. Initial management with colonoscopic decompression was attempted but proved unsuccessful, with persistent abdominal distension and tympani necessitating surgical intervention. Intraoperative findings confirmed a dolichosigmoid with mesenterico-axial volvulus, requiring sigmoid colon resection with creation of a double-barrel stoma using the Bouilly-Volkmann technique. Pathological examination of the resected specimen (30 cm in length, 491 g in weight) revealed flattened, hemorrhagic mucosa with vascular congestion and edema (Fig. 3). Microscopic analysis identified numerous Schistosoma eggs of varying morphology (Fig. 4A and B) within the submucosa and muscular layers, accompanied by granulomatous inflammation (Fig. 4C) and dense eosinophilic infiltrates. Chronic changes including eosinophilic microabscesses and calcified eggs were also noted (Fig. 5).

The patient's postoperative course was uncomplicated, with discharge on postoperative day 4. Subsequent parasitological examination of stool samples confirmed *Schistosoma mansoni* infection, for which he received praziquantel (40 mg/kg single dose). At 2-week follow-up, the patient reported only mild, intermittent abdominal pain with otherwise excellent functional recovery.

## Discussion

Sigmoid volvulus represents one of the most frequent causes of acute large bowel obstruction, ranking among the top three etiologies [1, 2]. Multiple predisposing factors have been identified, including congenital anatomical abnormalities such as intestinal malrotation and Hirschsprung disease, physiological states like pregnancy, and various clinical conditions including neuropsychiatric disorders (particularly Parkinson's disease and multiple sclerosis), chronic constipation with laxative dependence, and certain myopathies including Duchenne muscular dystrophy [1, 2]. Despite these well-established risk factors, the co-occurrence of sigmoid volvulus with schistosomiasis remains exceptionally rare in clinical practice, with no clearly demonstrated pathophysiological relationship between these two conditions [3, 7].

Schistosomiasis (bilharziasis) represents a major global health challenge as the world's second most prevalent parasitic disease after malaria, with endemic foci persisting across sub-Saharan Africa, South America, and parts of Asia [3, 7]. In non-endemic regions such as Tunisia, cases predominantly occur among immigrant populations or returning travelers from endemic zones. While *S. mansoni* and *S. japonicum* are the species most frequently associated with gastrointestinal involvement, colonic manifestations develop less commonly than the classic urinary tract pathology [7, 8]. The clinical presentation varies remarkably from completely asymptomatic infection to severe complications including intestinal obstruction, typically accompanied by nonspecific symptoms such as chronic diarrhea, recurrent abdominal pain, and nutritional deficiencies [7, 8]. Endoscopic evaluation often reveals a spectrum of mucosal abnormalities ranging from subtle edema to frank ulcerations and pseudopolyploid lesions [7, 8].

Diagnostic approaches have evolved significantly, with current options including traditional microscopic stool examination for parasite ova [9, 10], various serological assays (though these cannot reliably distinguish between active and past infections) [9, 10], and more recently developed molecular techniques such as polymerase chain reaction which demonstrates excellent sensitivity (94.4%) and specificity (99.9%) in urine samples [9, 10]. Additional diagnostic modalities include detection of circulating parasite-derived antigens which can provide evidence of active infection [9, 10].

To date, the medical literature contains only six well-documented cases of sigmoid volvulus unexpectedly revealing underlying schistosomal infection (Table 1) [3–5, 11–13]. Our case represents the seventh such occurrence and highlights several important clinical lessons. In all reported instances, including ours, the parasitic infection was entirely unsuspected prior to the acute volvulus event, emphasizing the protean manifestations of chronic schistosomiasis. Histopathological examination remains the gold standard for definitive diagnosis, typically demonstrating the characteristic parasitic eggs embedded within the submucosa associated with granulomatous inflammation and marked eosinophilic infiltration [14]. Over time, chronic infection leads to progressive fibrotic changes in the intestinal wall with eventual calcification of retained eggs [14]. The management of such cases requires both acute surgical intervention for the volvulus and subsequent antiparasitic therapy, underscoring the importance of multidisciplinary care.

**Table 1:** Cases of concomitant chronic schistosomiasis and sigmoid volvulus reported in the literature.

**Author**	**Year**	**Age / gender**	**Patients' nationalities**	**Treatment**
Mourra N [11]	2006	40 / Male	Angolan	Sigmoid colectomy and praziquantel
Buchwald P [4]	2010	33 / Male	Togolese	Sigmoid colectomy and praziquantel
George SA [12]	2015	20 / Male	Kuwaiti (Travel history to Egypt)	Sigmoid colectomy and praziquantel
Krog AD [5]	2018	72 / Male	Danish (Travel history to Botswana)	Sigmoid colectomy and praziquantel
Ilyas G [3]	2019	20 / Male	Egyptian	Sigmoid colectomy with praziquantel
Althumali A [13]	2022	21 / Male	Sudanese	Sigmoid colectomy and praziquantel
Our case	2025	22 / Male	Guinean	Sigmoid colectomy and praziquantel

## Conclusion

In non-endemic regions like Tunisia, surgeons should suspect colonic schistosomiasis in patients with travel or immigration history from endemic areas. The rare association with sigmoid volvulus highlights the need for awareness. Since symptoms may be absent, histological confirmation is crucial. Early detection prevents misdiagnosis, requiring collaboration between surgeons and pathologists. Routine histopathology of all colectomy specimens is essential, even if normal-looking. Prompt anti-helminthic treatment improves outcomes and reduces complications.

## Data Availability

All data are available in the manuscript. Further enquiries can be directed to the corresponding author.
